# DNA Extraction from Dry Museum Beetles without Conferring External Morphological Damage

**DOI:** 10.1371/journal.pone.0000272

**Published:** 2007-03-07

**Authors:** M. Thomas P. Gilbert, Wendy Moore, Linea Melchior, Michael Worobey

**Affiliations:** 1 Department of Ecology and Evolutionary Biology, The University of Arizona, Tucson, Arizona, United States of America; 2 Ancient DNA and Evolution, The Niels Bohr Institute, University of Copenhagen, Copenhagen, Denmark; 3 Department of Entomology, California Academy of Sciences, San Francisco, California, United States of America; 4 Research Laboratory, Institute of Forensic Medicine, The University of Copenhagen, Copenhagen, Denmark; Max Planck Institute for Evolutionary Anthropology, Germany

## Abstract

**Background:**

A large number of dry-preserved insect specimens exist in collections around the world that might be useful for genetic analyses. However, until now, the recovery of nucleic acids from such specimens has involved at least the partial destruction of the specimen. This is clearly undesirable when dealing with rare species or otherwise important specimens, such as type specimens.

**Methodology:**

We describe a method for the extraction of PCR-amplifiable mitochondrial and nuclear DNA from dry insects without causing external morphological damage. Using PCR to amplify ≈220 bp of the mitochondrial gene cytochrome *c* oxidase I, and 250–345 bp fragments of the multi-copy, nuclear 28s ribosomal DNA gene, we demonstrate the efficacy of this method on beetles collected up to 50 years ago.

**Conclusions:**

This method offers a means of obtaining useful genetic information from rare insects without conferring external morphological damage.

## Introduction

Dry insect specimens are commonly held in entomology collections, constituting over 900,000 described species and an unknown number of undescribed species. DNA sequence data provides valuable information for both phylogenetic inference [1] and taxonomic identification [2]. Since DNA is known to degrade post-mortem as a function of heat and time [3] molecular-based studies are largely limited to recently collected samples preserved specifically for molecular work. Naturally this prevents the use of specimens of a large number species that are now either extinct, or that have not been collected recently and preserved specifically for molecular work.

Many insect species are known from few (often one) individuals collected many years ago. Since standard DNA extraction methods involve at least partial specimen destruction [4], [5] they are unattractive for use on rare or otherwise important specimens (e.g. type specimens, voucher specimens). To overcome these limitations we have developed a method of extracting DNA without conferring visible, external morphological damage to such specimens.

## Materials and Methods

Fourteen dry specimens of the carabid beetle subfamily Paussinae, which had been collected between 2 and 94 years ago, were chosen for DNA analysis (Table 1). Full precautions were taken to prevent contaminating the samples with previously amplified DNA. Specifically, DNA extractions and subsequent manipulation prior to Polymerase Chain Reaction (PCR) amplification were conducted in a laboratory dedicated to research on samples that contain low amounts of DNA. Furthermore, this laboratory is physically isolated from the laboratory where post-PCR work is performed. To ensure the sequence accuracy, DNA was sequenced from multiple PCR amplifications for each sample.

**Table 1:** Details of specimens investigated in the study

Species	Pinned	Body length (mm)	Geographic Origin	Year Collected	Amplifiable nuDNA (Size bp)	Maximum dilution (nuDNA)	Amplifiable mtDNA	Accession ID (nuDNA/mtDNA)
*Granulopaussus graulatus*	No	3	South Africa	1910	No	n/a	No	n/a
*Edaphopaussus dissimulator*	No	8	Liberia	1952	Yes (255)	10	No	EF424229 (nu)
*Platyrhopalopsis mellei*	Yes	10	India	1966	Yes (258)	10	Yes	EF424233 (nu)
								EF424244 (mt)
*Itamus* sp.	Yes	12	Sri Lanka	1968	Yes (280)	100	No	EF424238 (nu)
*Anentmetus sp*	Yes	11	Sri Lanka	1970	Yes (332)	100	Yes	EF424237 (nu)
								EF424242 (mt)
*Heteropaussus lujae*	Yes	8	Cameroon	1974	Yes (304)	100	No	EF424228 (nu)
*Heteropaussus hastatus.*	Yes	8	South Africa	1980	No	n/a	Yes	EF424248 (mt)
*Afrozaena luteus*	Yes	7	Cameroon	1980	Yes (281)	10	Yes	EF424232 (nu)
								EF424243 (mt)
*Physea lapites*	Yes	6	Mexico	1984	No	n/a	Yes	EF424241 (mt)
*Entomoantyx cyanipennis*	No	4	Costa Rica	1992	Yes (345)	10	Yes	EF424230 (nu)
								EF424239 (mt)
*Platycerozaena magna*	Yes	18	Ecuador	1992	Yes (318)	10	Yes	EF424231 (nu)
								EF424240 (mt)
*Eohomopterus aequatoriensis*	No	5	Ecuador	1996	Yes (332)	100	Yes	EF424234 (nu)
								EF424245 (mt)
*Protopaussus* sp. A	No	5	China	2001	Yes (264)	100	Yes	EF424235 (nu)
								EF424246 (mt)
*Protopaussus* sp. B	No	5	Thailand	2002	Yes (250)	10	Yes	EF424236 (nu)
								EF424247 (mt)

Whole specimens were placed in 2 ml Eppendorf Biopur tubes, fully immersed in digestion buffer (volume dependent on specimen size, in our experiments 0.5–0.7 ml), and incubated overnight at 55°C with gentle agitation. The buffer was modified from Pfeiffer *et al*. [6] and consisted of 3 mM CaCl_2_, 2%sodium dodecyl sulphate (SDS), 40 mM dithiotreitol (DTT), 250 µg/ml proteinase K, 100 mM Tris buffer pH 8 and 100 mM NaCl (final concentrations). After incubating with gentle agitation for 16–20 hours, specimens were removed from the buffer, placed in 100%EtOH for 2–4 hours to stop further digestion, air-dried, and replaced in their collections. Nucleic acids were purified from the digestion buffer using a phenol:phenol:chloroform extraction [7] followed by isopropanol precipitation. Specifically, 20 µg glycogen (or 5 µl glycoblue, Ambion), 0.6 volumes 100%isopropanol and 0.1 volumes 3M Sodium acetate pH 5.2 were added, the mixture was immediately vortexed gently, and centrifuged at room temperature at maximum speed for 25 minutes to pellet the nucleic acids. The liquid was then removed and the pellet was washed twice in 1.5 ml ice-cold 85%ethanol, allowed to air-dry at 65°C, and resuspended in 100 µl molecular biology grade H_2_O or TE buffer.

The presence of amplifiable mitochondrial (mtDNA) and multi-copy nuclear DNA (nuDNA) in the extract was assayed through PCR. MtDNA was assayed through the amplification of a short (220 bp) fragment of the cytochrome *c* oxidase I (CO1) gene using primers ShortF (5′ CAATTTCCAAATCCNCCAAT) and ShortR (5′ GGTCAACAAATCATAAAGATATTGGAA, annealing temperature 50°C). The targeted fragment lies within the so-called ‘DNA Barcoding’ region chosen by the Consortium for the Barcode of Life (CBOL) [8].

NuDNA was assayed through the amplification of a fragment of 28s ribosomal DNA (250–345 bp depending on species). Each extract was assayed with 3 primer sets (to account for suspected sequence variation over the forward primer binding sites). The forward primers D3F (5′AGG ACC CGT CTT GAA ACA CGG, annealing temperature 54°C), D3Fb (5′ CACGGACCAGGGAGTCTAGCAT, annealing temperature 50°C) or D3Fc (5′ GGA CCA GGG AGT CTA GCA T, annealing temperature 48°C) were paired with reverse primer D3R (5′GCA TAG TTC ACC ATC TTT C).

Each 25 µl PCR reaction contained 1 µl extracted DNA, 0.1 µl Platinum Taq (Invitrogen), 0.1 µl 25 mM mixed dNTPs, 2.5 µl 10x PCR buffer, 0.3 µM each primer, and 2.5 mM MgCl_2_, and was cycled 40 times. PCR was tested on the original extract plus 10-and 100-fold dilutions, to briefly investigate the quantity of useable DNA extracted from each specimen. All amplified PCR products were purified using QIAquick spin columns (Qiagen) and sequenced several times using an ABI 3730xl capillary sequencer (Applied Biosystems), in both directions, using BigDye Terminator V 3.1 chemistry (Applied Biosystems).

## Results

In total, DNA could be recovered from 13 of the 14 specimens examined. Specifically, NuDNA was successfully extracted and sequenced from 11/14 samples, the exceptions being the oldest sample, collected 94 years ago, and two more recent samples (Table 1). Mitochondrial DNA was successfully extracted and sequenced from 10/14 samples. No PCR or extraction blanks exhibited evidence of contamination with beetle DNA. To ensure our results were not contaminants, we aligned our sequences with those from 70 other paussine beetles, and their identity was confirmed phylogenetically.

**Figure 1:**
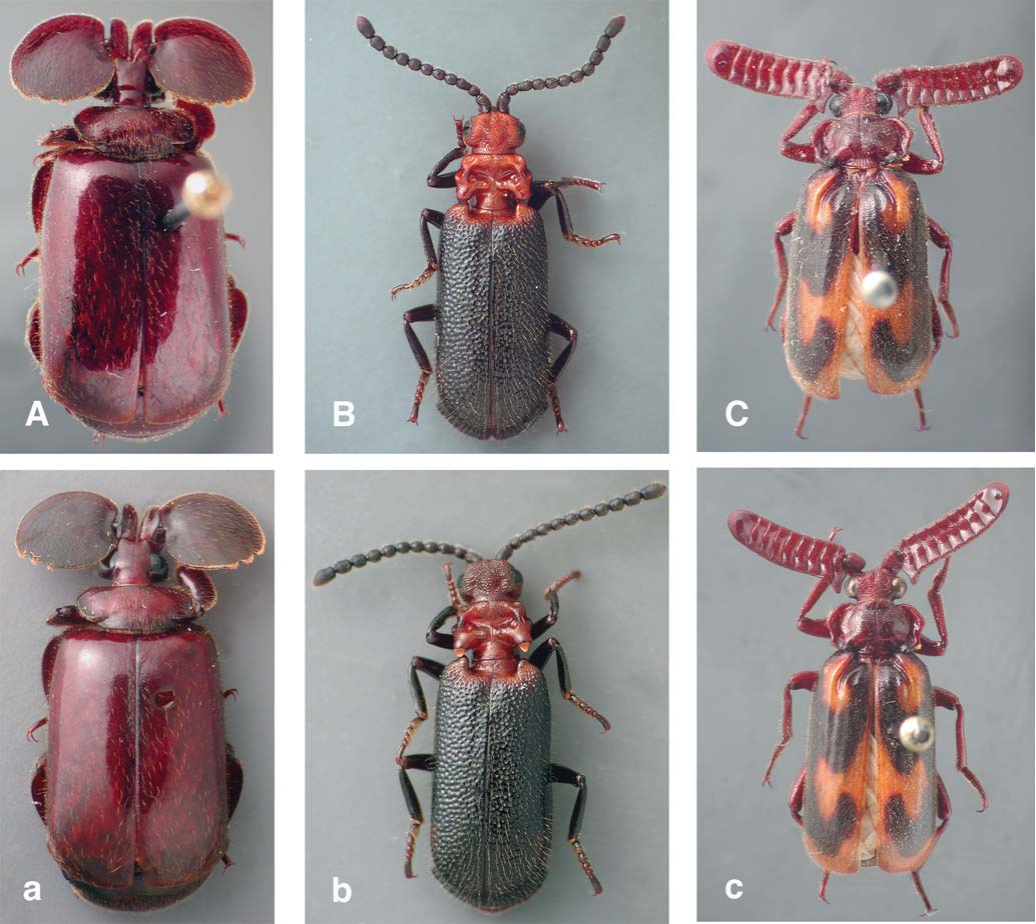
Samples pre-and post-extraction. Photographs of three beetle specimens before (A,B,C) and after (a,b,c) overnight treatment in the extraction buffer. The specimens are as follows: A/a *Platyrhopalopsis mellei* (collected in 1966), B/b *Protopaussus* sp. B (collected in 2002), *C/c Heteropaussus hastatus* (collected in 1980).

As demonstrated in Fig. 1, the samples exhibit no significant external change/damage post extraction, thus validating the use of this method on important specimens. In addition, sufficient DNA was retrieved to enable us to undertake PCRs at 10-(and often 100-) fold dilutions of the extract, theoretically providing enough DNA for 1,000–10,000 PCRs per sample.

## Discussion

### Discussion and Caveats

Although one previous study has described a non-destructive extraction method for insect specimens [9], in fact the described method requires physical puncturing, thus damage, of the exoskeleton prior to digestion. Thus in contrast to our method, the previous method is not truly non-destructive. Our method parallels a conceptually similar approach recently used to extract DNA from museum samples of mammal teeth [10]. In that study, the authors argue that the extraction buffer enters the sample and liberates DNA through dentinal tubules that perforate the teeth. In beetles we speculate the buffer liberates DNA through the mouth, anus, spiracles, and possibly through areas of thin cuticle between sclerites, ectodermal glands and possibly broken setae. DNA is almost certainly also released through the man-made opening in the left elytron and pterothorax of pinned beetle specimens. Dissection of the thorax and abdomen of one treated specimen revealed partial digestion of the internal tissue.

In the case of extremely precious material, we urge scientists to consider using our extraction method on a single leg dissected from the specimen. After extraction the undamaged leg can be replaced on a card with the pinned specimen. If one considers the virtual certainty that DNA extraction and other molecular protocols will improve in the future, limiting the extraction to one leg would ensure that undigested tissue remains in the specimen for future use.

In the present study, failure to amplify nuDNA and mtDNA from the oldest specimen is likely due to post-mortem degradation of DNA to sub-amplifiable levels [3]. The reason as to why two much younger specimens did not yield nuDNA is unknown. It is possible that the unknown preservation and storage conditions of these samples may have degraded all of the DNA to sub-amplifiable quality. Alternatively we cannot rule out that sequence divergence in these samples at primer binding sites may have prevented amplification. Lastly it might be argued that the extraction buffer itself is detrimental to DNA. Although we have not explicitly tested this, the buffer is predominantly modified from other DNA extraction buffers through an increase in the detergent (SDS), which would not have a degradative effect on the DNA [7].

We find it surprising however that three samples that yielded amplifiable nuDNA did not yield amplifiable mtDNA (for example, see *Physea lapites,* Table 1). Studies that investigate old and degraded DNA have as a rule demonstrated that the inherent high ratio of mitochondrial to nuclear template molecules will result in mtDNA remaining PCR-amplifiable for a longer period of time than nuDNA in any specific sample [3]. Furthermore, we note that the mtDNA fragments are smaller than those nuDNA amplified from the particular samples. As with the failed attempt to amplify nuDNA from the two relatively recent specimens, an explanation may simply be that sequence divergence in the problematic samples at the primer binding sites may have prevented amplification.

DNA degradation in dead tissue correlates with a number of factors including the presence of free water, oxygen, heat and time since death [3]. Insect specimens are usually killed with ethyl acetate, ethyl alcohol, formalin or cyanide depending upon the taxon, the collection method (e.g. hand collection, malaise traps, pitfall traps), and the preference of the collector. Specimen labels usually do not include details such as which killing agent was used, and other aspects surrounding post-mortem conditions including the amount of time the specimen was exposed to the killing agent. Therefore, we caution that successful DNA extraction and amplification cannot necessarily be expected from all such specimens: it is possible that varying collection and storage conditions may give rise to prolonged or reduced DNA survival.

As such, the maximum DNA fragment size that will be amplifiable in each sample will depend on how degraded the DNA is prior to the analysis. For similar reasons, in our study quantification of the DNA in the extracts using quantitative real-time PCR or alternative techniques would provide little useful data to enable other researchers to decide if the method is suitable for their use, as the level of DNA in other extracts will depend on the specific sample, the collection method, and its history since collection, rather than the extraction method. Furthermore, we acknowledge that the targets of this study, chosen for their phylogenetic applicability (the data has been used in Moore, *in prep*, Phylogeny of the Flanged Bombardier Beetles (Carabidae: Paussinae) based on DNA sequence data) are multicopy genes, thus rendering them potentially easier to PCR amplify than single copy nuclear genes. Therefore, as with all studies that use old specimens, researchers will need to customise their PCR assays to the condition of the DNA. In addition, as with all other studies on sources of degraded DNA, we caution researchers to be aware of possible contaminants on samples that may affect the analyses–chiefly among which is previously amplified DNA. We also caution that our study samples are beetles, which have fairly robust exoskeletons, and it is possible that although this method can be extended to other insects, more fragile specimens may undergo more significant morphological change (although probably no worse than if they are treated with conventional methods). Furthermore, we acknowledge that our results are based on a single taxon within the beetles, all samples are derived from paussine species. However, unless our results can be attributed to the fact that DNA escapes from the specimens in solution through paussine-specific features of beetle anatomy, it is unlikely that the method will not be suitable for use on other beetles. In contrast, we believe that it is likely that the success of this approach will not be limited to beetles, and that it may prove useful on many other arthropods including arachnids and crustaceans.
